# The prevalence of preterm and low birth weight infants among migrant women in the Pearl River Delta region, China: a population-based birth cohort study

**DOI:** 10.1186/s12889-024-18667-8

**Published:** 2024-04-26

**Authors:** Lulu Xie, Zhijiang Liang, Xionghu Wang, Xianqiong Luo

**Affiliations:** 1 Department of Pediatrics, Guangdong Women and Children Hospital, Guangzhou, 511442 China; 2 Department of Public Health, Guangdong Women and Children Hospital, Guangzhou, 511442 China; 3 Department of Health Care, Guangdong Women and Children Hospital, Guangzhou, 511442 China

**Keywords:** Preterm birth, Low birth weight, Migrant women, Pearl river delta region, Healthy migrant effect

## Abstract

**Background:**

The existing literature evaluating the association between neonatal morbidity and migrant status presents contradictory results. The purpose of this study was to compare the risk of preterm birth (PTB) and low birth weight (LBW) among newborns from local and migrant women in China’s Pearl River Delta (PRD) region.

**Methods:**

In this observational population-based study, we included all live singleton deliveries from PRD region local women and migrant women. Data were sourced from the Guangdong Medical Birth Registry Information System between Jan 1, 2014, and Dec 31, 2020. Women were categorized into three groups by maternal migrant status: local women from PRD region, migrant women from Guangdong province or from other provinces. The outcome variables that were examined included two adverse birth outcomes: PTB and LBW. The association between the risk of PTB and LBW and maternal migrant status was assessed using logistic regression.

**Results:**

During 2014–2020, 5,219,133 single live deliveries were recorded, corresponding 13.22% to local women and the rest to migrant women coming from Guangdong (53.51%) and other provinces (33.26%). PTB prevalence was highest among local women (5.79%), followed by migrant women from Guangdong (5.29%), and the lowest among migrants from other provinces (4.95%). This association did not change after including maternal age, infant sex, delivery mode, and birth season in the models. Compared to local women, migrant women from other provinces had a lower risk of LBW (4.00% vs. 4.98%, *P* < 0.001). The prevalence of PTB and LBW was higher among local women than migrants. The odds of delivery PTB and LBW were higher for women who were age ≥ 35. Among the three maternal migration groups, the age-LBW association displayed a typical U-shaped pattern, with those in the youngest (16–24 years) and oldest (≥ 35) age categories exhibiting the higher odds of delivering a LBW neonate. With respect to infant sex, the prevalence of PTB was significantly higher in males than females among the three maternal migration groups. An opposite trend was found for LBW, and the prevalence of LBW was higher in females among the three maternal migration groups.

**Conclusion:**

The findings of this study contribute to the understanding of the epidemiology of PTB and LBW among migrant women. Our study suggests that it is the health and robust nature of migrant mothers that predisposes them to better birth outcomes. It is important to recognize that the results of this study, while supportive of the healthy migrant effect, cannot be considered definitive without some exploration of motivation for moving and changes in lifestyle postmigration.

## Background

With an increasing number of babies born to migrant parents, over the last three or four decades, there has been significant interest in assessing the association between maternal migrant status and birth outcomes. Migrant women frequently initiate the mobility process at a childbearing age, irrespective of individual motivations for leaving their countries [1]. Previous studies have compared perinatal outcomes between local and migrant women with mixed results focusing, like most other studies on the topic worldwide, on preterm birth (PTB) and low birth weight (LBW). When comparing the risks between local and migrant women of having a PTB, or an LBW infant, some studies gathered evidence suggesting that migrants had more adverse outcomes [2–6] while others found no difference or better outcomes for migrants [7–11].

The existing literature evaluating the association between neonatal morbidity and maternal migrant status presents contradictory results. Migrant status has often been seen as a risk factor for adverse neonatal outcomes, such as PTB [3, 5], LBW [4], and perinatal morbidity and mortality [4, 12, 13]. In some instances, migrant groups have equivalent or better outcomes than local women. This is related to what has been called the “healthy migrant effect” or the “epidemiologic paradox” [14], which states only that healthy individuals migrate whereas the individuals at greatest risk stay behind. Most evidence about the healthy migrant effect comes from studies that focused on groups of Latino migrants in the USA [15]. Studies that focus on developing countries are limited with varied results. Some Chinese studies have investigated the “healthy migrant effect” [16]. Tong and Piotrowski found migrants to have better health when compared with the local urban population in Beijing [17]. However, recent research on the health status of Chinese migrants has yielded the opposite results [18].

Since the economic reform in the 1980s, China has experienced decades of rapid urbanization with large-scale rural-to-urban and west-to-east intercity migration [19]. China’s PRD region lies in the central southern coastal part of Guangdong. The PRD region covers nine cities and hosts over 90% of the migrants in Guangdong and accounts for more than 80% of its gross domestic product (GDP) [20, 21]. As one of the largest migrant-concentrated regions in China, the PRD region received approximately 51.99 million rural migrant workers in China [22], providing an opportunity for us to examine birth outcomes among migrant women. In 2021, Tang et al. first measured the effect of migrant status on the likelihood of high-risk pregnancies in China [14]. Estimates of the burden of PTB and LBW in migrant women are needed to understand the epidemiology of this condition because data are sparse and incomplete in China’s PRD region. The purpose of this study was to explore the associations between maternal migrant status and adverse birth outcomes.

## Methods

### Study design and population

This is a population-based observational study of all live singleton births to local and migrant women using a birth certificate database in the state of the PRD region, between January 1, 2014, and December 31,2020. We accessed data from the Guangdong Women and Children Health Information System. The available information includes region of maternal birth, date of birth, infant gender, gestational age at birth, birth weight, maternal age, and delivery mode. All these data are filled in system by the hospital staff or midwives according to the babies’ medical records, which are also checked by obstetricians. We excluded birth records with missing individual information or incorrect data: (1) births with missing gestational age or gestational age < 20 weeks or > 43 weeks. (2) Births with missing birth weight or birth weight < 500 g or > 5,000 g. We also excluded non-Chinese women or women without a delivery date. After these exclusions, this study included births to migrant women (*n* = 4,529,040) and local women (*n* = 690,093) giving birth in the PRD region in the period 2014–2020.

### Migrant status

Guided by our literature review and the resident registration system in China [23], we defined a “migrant” as a person who has settled permanently or temporarily in PRD region other than his/her own household registration in China. Women were categorized into the three following groups by maternal migrant status: (1) Local women from PRD region (Group 1): registered in the PRD region and delivered in the PRD region; (2) Migrant women from Guangdong province (Group 2): registered in another region of Guangdong and delivered in the PRD region; (3) Migrant women from other provinces (Group 3): registered in another province and delivered in the PRD region. We then calculated the proportion of PTB and LBW for each maternal migration group.

### The PRD region

Guangdong Province, has 21 cities, grouped into four regions according to their geographical location and economic conditions: the PRD, Eastern Guangdong, Western Guangdong, and Northern Guangdong. The PRD region includes Guangzhou, Shenzhen, Foshan, Dongguan, Zhuhai, Zhongshan, Jiangmen, Zhaoqing and Huizhou (Data sources: http://www.gd.gov.cn/).

### Outcome variables

Live birth refers to the complete expulsion or extraction from its mother of a product of conception, irrespective of the duration of the pregnancy [24]. In accordance with criteria recommended by the World Health Organization (WHO), PTB is defined as all births less than 37 whole weeks of gestation or fewer than 259 days since the first day of a woman’s last menstrual period [25], and LBW is a weight at birth that is less than 2500 g (up to and including 2499 g) [26]. The outcome variables of this study were PTB and LBW based on gestational age, PTB can be subdivided as follows: extremely PTB (< 28 completed weeks of gestation), very PTB (28 ≥ to < 32 weeks), or moderately PTB (32 ≥ to < 37 weeks) [27]. Infants with birth weight < 2500 g are further categorized into LBW, 1500–2499 g; very LBW, 1000–1499 g; and extremely LBW < 1000 g [26]. Maternal age at delivery was categorized into three groups: 16–24, 25–29,30–34,35–39, and 40–50 years old.

### Statistical analysis

Statistical analyses were conducted using IBM SPSS statistical software for Windows (Version 26.0. Armonk, NY: IBM Corp.). Mean gestational age and mean birth weight are expressed as the mean standard deviation (± SD), and categorical variables are expressed as frequencies and percentages. The chi-squared test was used for categorical data, and the Student’s t test, and Kruskal-Wallis H test were used to compare three independent samples. Multivariable logistic regression was used to run crude and adjusted models to assess the association of different immigrant-status mother groups with preterm birth and low-birth-weight. In the adjusted models we included four confounding factors: maternal age, infant sex, delivery mode, and birth season. Adjusted odds ratios (ORs) and 95% confidence intervals were calculated. Adjusted odds ratios (ORs) were calculated with a 95% confidence interval. Probability levels (P value) < 0.05 were considered significant.

## Results

During 2014–2020, 5,377,162 live births were born in the PRD region, Guangdong. We excluded 958 births with a gestational age less than 28 weeks or higher than 43 weeks, birth weights less than 500 g or higher than 5000 g, and 149,127 multiple births, leaving 5,219,133 births (97.06%) for the final analysis. During the period analyzed 5,219,133 single live deliveries were recorded, corresponding 13.22% to local women and the rest to migrant women coming from Guangdong (53.51%) and other provinces (33.26%).

Table 1 presents the demographic characteristics of women who delivered infants in the PRD region between 2014 and 2020 by maternal migrant status. As illustrated in Table 1, the total proportions of mothers aged 16–24, 25–29,30–34,35–39, and 40–50 years old were 20.58%, 37.75%,27.74%,11.38% and 2.45%, respectively. A maternal age of 16–24 years old was frequent for migrant women from other provinces (23.52%), followed by migrant women from Guangdong (20.69%) and local women (12.76%). Migrant women from Guangdong and other provinces were less likely to have a caesarean delivery (30.25% and 34.32%, respectively) than local women (35.15%). There were more births occurring in autumn and summer than in winter and spring among the three maternal groups. Infants of migrant mothers from other provinces had a higher mean gestational age and birth weight than those of local mothers and migrant mothers from Guangdong (all *P* < 0.001).

**Table 1:** Comparison of sociodemographic characteristics of migrants and local women giving birth to live singletons between 2014 and 2020 in PRD Region

Characteristic	Total *n* = 5,219,133	Group 1 *n* = 690,093	Group 2 *n* = 2,792,977	Group 3 *n* = 1,736,063	*P*-values ^a^
Maternal age, y (n, %)					
16 ∽ 24	1,074,234 (20.58)	88,041 (12.76)	577,940 (20.69)	408,253 (23.52)	< 0.001 ^A, B, C^
25 ∽ 29	1,970,479 (37.75)	256,200 (37.13)	1,092,518 (39.12)	621,761 (35.81)	
30 ∽ 34	1,447,580 (27.74)	224,487(32.53)	739,376(26.47)	483,717(27.86)	
35 ∽ 39	593,765 (11.38)	97,621(14.15)	315,716(11.30)	180,428(10.39)	
40 ∽ 50	127,682 (2.45)	23,421 (3.39)	65,683 (2.35)	38,578 (2.22)	
< 16 or missing data	5393 (0.10)	323 (0.05)	1744 (0.06)	3326 (0.19)	
Infant sex (n, %)					
male	2,792,678 (53.51)	368,491 (53.39)	1,495,098 (53.53)	929,089 (53.52)	< 0.001 ^A, B, C^
female	2,425,934 (46.48)	321,567 (46.60)	1,297,607 (46.46)	806,760 (46.47)	
Unknown	521 (0.01)	35 (0.01)	272 (0.01)	214 (0.01)	
Delivery mode (n, %)					
Vaginal delivery	3,535,799 (67.75)	447,506 (64.85)	1,947,974 (69.75)	1,140,319 (65.68)	< 0.001 ^A, B, C^
Cesarean	1,683,334 (32.25)	242,587 (35.15)	845,003 (30.25)	595,744 (34.32)	
Birth Season (n, %)					
Spring	1,175,442 (22.52)	163,745 (23.73)	630,546 (22.58)	381,151 (21.96)	< 0.001 ^A, B, C^
Summer	1,364,830 (26.15)	173,388 (25.13)	715,175 (25.61)	476,267 (27.43)	
Autumn	1,496,777 (28.68)	183,662 (26.61)	804,837 (28.81)	508,278 (29.28)	
Winter	1,182,084(22.65)	169,298 (24.53)	642,419 (23.00)	370,367 (21.33)	
Mean gestational age mean ± SD (wk)	38.74 ± 1.50	38.59 ± 1.50	38.73 ± 1.49	38.81 ± 1.51	< 0.001 ^A, B, C^
Mean birth weight mean ± SD (g)	3180.01 ± 440.70	3160.02 ± 439.31	3151.45 ± 432.63	3233.89 ± 449.08	< 0.001 ^A, B, C^

*Abbreviations* wk, gestational week; g: gram. Group 1: local women from PRD region; Group 2: migrant women from Guangdong province; Group 3: migrant women from other provinces. ^a^ P-values are comparisons between the local women and migrant women by use of Chi-square test. ^A^ Significant difference at 0.05 level between group 1 and group 2. ^B^ Significant difference at 0.05 level between group 1 and group 3. ^C^ Significant difference at 0.05 level between group 2 and group 3

The crude and adjusted associations between maternal migrant status and the risk of PTB and LBW are shown in Table 2. PTB prevalence was highest among local women (5.79%), followed by migrant women from Guangdong (5.29%), and the lowest among migrant women from other provinces (4.95%). This association did not change after including maternal age, infant sex, delivery mode, and birth season in the models. Compared to local women, migrant women from other provinces had a lower risk of LBW (4.00% vs. 4.98%, *P* < 0.001). The adjusted odds ratio was 0.792 (95% CI: 0.781, 0.802). In unadjusted and adjusted regression analyses, migrant women from Guangdong and women from other provinces had significantly lower odds of overall PTBs, extremely PTBs, very PTBs, and moderately PTBs. Similarly, migrant women from other provinces also had lower risk of overall LBW, LBW, very LBW, and extremely LBWs, but not statistically significant in overall LBW, very LBW, and LBW births in the adjusted model (adjusted OR = 1.001 [0.989–1.014], adjusted OR = 0.964 [0.921–1.009], adjusted OR = 1.011 [0.998–1.023], respectively).

**Table 2:** Crude and adjusted odds ratios with confidence intervals (95%CI) for PTB and LBW births by maternal migrant status

Outcomes	Group 1 *n* = 690,093	Group 2 *n* = 2,792,977	Group 3 *n* = 1,736,063	*P*-values ^a^
PTB, n (%)	39,986 (5.79)	147,697 (5.29)	85,860 (4.95)	
OR (95%CI)	1.00	0.908 (0.898, 0.918)	0.846 (0.836, 0.856)	< 0.001 ^A, B, C^
OR _adj_ (95%CI)	1.00	0.944 (0.933, 0.955)	0.869 (0.858, 0.879)	< 0.001 ^A, B, C^
< 28w, n (%)	523 (0.08)	1542 (0.06)	975 (0.06)	
OR (95%CI)	1.00	0.725 (0.656, 0.800)	0.734 (0.660, 0.817)	< 0.001 ^A, B, C^
OR _adj_ (95%CI)	1.00	0.755 (0.684, 0.834)	0.793 (0.713, 0.882)	0.006 ^A, B, C^
28 ∽ 31w, n (%)	3264 (0.47)	11,248 (0.40)	7837 (0.45)	
OR (95%CI)	1.00	0.847 (0.814, 0.881)	0.946 (0.908, 0.985)	< 0.001 ^A, B, C^
OR _adj_ (95%CI)	1.00	0.889 (0.855, 0.924)	0.982 (0.942, 1.023)	< 0.001 ^A, B, C^
32 ∽ 36w, n (%)	36,199 (5.24)	134,907 (4.83)	77,048 (4.44)	
OR (95%CI)	1.00	0.916 (0.905, 0.927)	0.839 (0.828, 0.849)	< 0.001 ^A, B, C^
OR _adj_ (95%CI)	1.00	0.952 (0.940, 0.963)	0.860 (0.849, 0.871)	< 0.001 ^A, B, C^
LBW, n%	34,384 (4.98)	137,262 (4.91)	69,473 (4.00)	
OR (95%CI)	1.00	0.986 (0.974, 0.998)	0.795 (0.785, 0.806)	< 0.001 ^A, B, C^
OR _adj_ (95%CI)	1.00	1.001 (0.989, 1.014)	0.792 (0.781, 0.802)	< 0.001 ^A, B, C^
500 ∽ 999 g, n%	641 (0.09)	1584 (0.05)	1044 (0.06)	
OR (95%CI)	1.00	0.610 (0.557, 0.669)	0.641 (0.581, 0.707)	< 0.001 ^A, B, C^
OR _adj_ (95%CI)	1.00	0.665 (0.607, 0.729)	0.698 (0.632, 0.770)	< 0.001 ^A, B, C^
1000 ∽ 1499 g, n%	2336 (0.34)	8560 (0.31)	5311 (0.31)	
OR (95%CI)	1.00	0.905 (0.864, 0.947)	0.895 (0.852, 0.939)	< 0.001 ^A, B, C^
OR _adj_ (95%CI)	1.00	0.964 (0.921, 1.009)	0.930 (0.885, 0.976)	< 0.001 ^A, B, C^
1500 ∽ 2499 g, n%	31,407 (4.55)	127,118 (4.55)	63,118 (3.63)	
OR (95%CI)	1.00	0.999 (0.987, 1.012)	0.791 (0.780, 0.802)	< 0.001 ^A, B, C^
OR _adj_ (95%CI)	1.00	1.011 (0.998, 1.023)	0.784 (0.773, 0.795)	< 0.001 ^A, B, C^

*Abbreviations* OR: Crude odds ratios; OR _adj_: adjusted odds ratios; CI: confidence intervals; Group 1: local women from PRD region; Group 2: migrant women from Guangdong province; Group 3: migrant women from other provinces. OR _adj_: Adjusted odds ratio for maternal age, infant sex, delivery mode, and birth season. Reference group: group 1 ^a^ P-values are comparisons between the local women and migrant women by use of Chi-square test. ^A^ Significant difference at 0.05 level between group 1 and group 2 ^B^ Significant difference at 0.05 level between group 1 and group 3 ^C^ Significant difference at 0.05 level between group 2 and group 3

Table 3 shows the effect of the maternal migrant status on the rate of PTB and LBW stratified by maternal age, infant sex, and delivery mode. The prevalence of PTB and LBW was higher among local women than migrants in the three maternal age groups. The odds of delivery PTB and LBW were higher for women aged from 40 to 50 among the three maternal migration groups. The highest rates of PTB and LBW were 10.1% and 7.02%, respectively, in the local mothers aged from 40 to 50. Among the three maternal migration groups, the age-LBW association displayed a typical U-shaped pattern, with those in the youngest (16–24 years) and oldest (40–50 years) age categories exhibiting higher odds of delivering an LBW neonate. With respect to infant sex, the prevalence of PTB was significantly higher in males than females among the three maternal migration groups. An opposite trend was found for LBW, and the prevalence of LBW was higher in females among the three maternal migration groups. With PTB or LBW as the outcome, migrant women from Guangdong and other provinces were less likely to receive cesarean compared with those who were born from the PRD region.

**Table 3:** The effect of the maternal migrant status on the rate of PTB and LBW births stratified by maternal age, infant sex, and delivery mode

variable	Maternal migrant status	PTB			LBW		
		*n*	%	OR _adj_ (95%CI)	*n*	%	OR _adj_ (95%CI)
By maternal age							
16 ∽ 24	Group 1	4592	5.22	1.00	5008	5.69	1.00
	Group 2	28,445	4.92	0.948 (0.918, 0.979)	31,342	5.42	0.960 (0.931, 0.990)
	Group 3	19,276	4.72	0.897 (0.867, 0.927)	18,792	4.60	0.796 (0.771, 0.822)
25 ∽ 29	Group 1	12,643	4.93	1.00	11,691	4.56	1.00
	Group 2	51,302	4.70	0.949 (0.930, 0.968)	50,190	4.59	1.007 (0.987, 1.028)
	Group 3	25,977	4.18	0.840 (0.822, 0.858)	21,195	3.41	0.738 (0.721, 0.755)
30 ∽ 34	Group 1	12,918	5.75	1.00	10,495	4.68	1.00
	Group 2	39,928	5.40	0.935(0.916, 0.954)	33,834	4.58	0.978 (0.956, 1.000)
	Group 3	24,583	5.08	0.877 (0.858, 0.896)	18,156	3.75	0.856 (0.776, 0.815)
35 ∽ 39	Group 1	7406	7.59	1.00	5494	5.63	1.00
	Group 2	21,965	6.06	0.911 (0.886,0.936)	17,280	5.47	0.971 (0.941,1.002)
	Group 3	12,185	6.75	0.882 (0.856,0.909)	8622	4.78	0.842 (0.813,0.871)
Male 40 ∽ 50	Group 1	2358	10.1	1.00	1644	7.02	1.00
	Group 2	5840	8.9	0.872 (0.829,0.917)	4394	6.69	0.950 (0.895,1.007)
	Group 3	3429	8.9	0.871 (0.825,0.921)	2335	6.05	0.853 (0.799,0.911)
Female By infant sex							
male	Group 1	23,297	6.32	1.00	16,780	4.55	1.00
	Group 2	86,880	5.81	0.946 (0.932, 0.960)	65,950	4.41	0.981 (0.964, 0.998)
	Group 3	49,783	5.36	0.860 (0.846, 0.874)	33,615	3.62	0.784 (0.769, 0.799)
female	Group 1	16,658	5.18	1.00	17,578	5.47	1.00
	Group 2	60,785	4.68	0.943 (0.926, 0.960)	71,282	5.49	1.023 (1.005, 1.040)
	Group 3	36,053	4.47	0.882 (0.865, 0.898)	35,835	4.44	0.800 (0.785, 0.815)
By delivery mode							
Vaginal delivery	Group 1	21,367	4.77	1.00	18,946	4.23	1.00
	Group 2	89,587	4.60	0.973 (0.959, 0.988)	85,009	4.36	1.024 (1.007, 1.040)
	Group 3	47,026	4.12	0.872 (0.858, 0.887)	39,080	3.43	0.793 (0.779, 0.807)
Cesarean	Group 1	18,619	7.68	1.00	15,438	6.36	1.00
	Group 2	58,110	6.88	0.906 (0.891, 0.922)	52,253	6.18	0.971 (0.953, 0.989)
	Group 3	38,834	6.52	0.866 (0.850, 0.882)	30,393	5.10	0.791 (0.775, 0.807)

*Abbreviations* OR _adj_: adjusted odds ratios; CI: confidence intervals; Group 1: local women from PRD region; Group 2: migrant women from Guangdong province; Group 3: migrant women from other provinces. OR _adj_: Adjusted odds ratio for maternal migrant status. Reference group: Group 1

## Discussion

The results of this study involving a large cohort of live births provide a clear description of PTB and LBW discordance between local and migrant women in the PRD region in Guangdong Province. Our study analyzed a total of 5,219,133 single live births to reveal the prevalence of PTB and LBW between local women and migrant women in the PRD region from 2014 to 2020. Our main findings show that, in comparison to local women, migrant women from Guangdong and other provinces have significantly lower odds of PTB and LBW. The present research findings indicated that migrant women had a clearly positive status of birth outcomes. This study reveals the differences in PTB and LBW between local and migrant women in the PRD region, and provides more recent data about the reproductive status of pregnant women in the PRD region.

China has the largest group of rural-to-urban floating women worldwide, most of whom are of childbearing age [28]. As one of the largest migrant-concentrated regions in China, the PRD region provided a good site to examine birth outcomes among migrant women. In southern China, the PRD region had a better economic situation, education conditions and nutritional support than the non-PRD regions [29]. In our study, 86.78% were migrant women over the last seven years. The ratio of migrant women who gave birth in PRD to those local women is approximately 6.5 to 1. This study showed that in general, there are significant differences in the perinatal outcome among local and migrant childbearing women. We found that maternal age over 35 years and cesarean section rates were higher in local women, while the proportion of women aged 16–24 years was significantly higher among migrants, especially the migrant women from other provinces. This may be explained by the predominant type of migration to the PRD region, with migrants being mostly laborers. It is possible to associate the higher maternal age in local patients with the education levels of these women, the time they spent on their education, and whether they had a profession [30].

Previous studies have shown that perinatal outcomes, including PTB and LBW, vary by country within regions. Our study revealed that the rates of PTB and LBW in the PRD region were relatively low, compared with those in other countries worldwide. During the period from 2014 to 2020, the overall PTB rate of 5.24% in the PRD region was at a relatively lower level compared with the global PTB rate ranging from 5% in northern European countries to 18% in African countries [31]. It is also lower than the weighted national incidence of 6.7% in China during 2015–2016 [32]. The rates of LBW in the PRD region (4.62%) were still lower than those of some developed countries, such as the USA (8%), Australia (7%), the UK (8%), Canada (6%) and Japan (8%) [33].

The international literature shows conflicting evidence regarding perinatal outcomes among migrant women compared to the majority population. Some studies have identified migration status as a risk factor [4, 34–36]. However, other studies have found that migration is a protective factor regarding perinatal outcomes [9, 37–39]. In our study, the birth outcome PTB and LBW rates of migrant women were lower than those of local women. Previous studies comparing migrant and nonmigrant birth outcomes point to a related explanatory hypothesis: the “healthy migrant effect” [40, 41]: women who migrate are particularly healthy on average, and remain so for some time despite socioeconomic disadvantages they face in the host country, thanks in part to strong familial ties. To date, most evidence about the healthy migrant effect on pregnancy outcomes has been collected in studies from the USA; the health disadvantage of migrants or no difference is a more common finding in European countries [15]. Migrant women enjoy health advantages to a certain extent because healthier people are more likely to migrate [42]. We can also theorize that the migrant women are generally in good health, have the economic ability to access health care services and are representative of a healthy self-selected migrant, which could be the true cause of the favorable birth outcomes of these migrants. A “healthy migrant effect” was found and maintained after controlling for various demographic variables. After adjustment, status as migrants were protective in our study. Our study lends support to the “healthy migrant effect”. This suggests that healthy individuals are more likely to migrate than their less-healthy compatriots and that these individuals opt for regions with higher incomes, as in this study. Those mothers who are healthy and able to move, regardless of whether the movement is from one country to another or from one region to another, have healthier babies.

The better outcome of birth weight and LBW outcomes, may be related to these women representing a selected group of migrants. Maternal body composition is one of the most important factors that accounts for geographical variation in neonatal outcomes. The differences in the risk of LBW may be due to differences in genetics or other factors, which were not measured in our study. Overall, people living in northern China had a mean BMI more than 2 kg/m² higher than their counterparts in southern China [43]. Likewise, there was more than a threefold difference in obesity prevalence between northern China and southern China [43]. It has been postulated that nutritional transitions might be at different stages in different parts of China [44]. This is supported by the results of the present study since mothers originating in northern China have a higher BMI than women from southern China and seem more likely to have heavier offspring. Other factors, such as maternal diet, physical activity, illness, and social class, vary across different migrant groups, and these along with genetic mechanisms may explain differences in neonatal outcomes among women of migrant origin and PRD region-born women. As this study found evidence for advantageous birth outcomes birth weight of migrant women, it lends weight to the proposition that the disparities in outcomes between local women and migrant women may be more reflective of the individual characteristics of the women than the sociocultural context of China.

Local women were at higher risk of cesarean delivery compared with migrant women. This result stands in contrast to those reported in other studies in which migrant women were found to have higher rates of cesarean [45, 46]. Suggested protective factors for cesarean delivery included a healthy migrant effect, preference for a vaginal birth, a healthier lifestyle, younger mothers, and the use of fewer interventions during childbirth [46]. Research suggests that pathways leading to cesarean births in migrants are complex, and they are likely to involve a combination of factors related to migrant women’s physical and psychological health, their social and cultural context and the quality of their maternity care [45]. There is insufficient evidence in our study to explain the observed differences.

The results show that mother’s age has a U-shaped relationship with the LBW rate among the three maternal migration groups. The risk of PTB was higher for women aged from 40 to 50 years in all three maternal migration groups and subsequently decreased in the 16–24 year old group and 25–34 year old group. In our study, the prevalence of PTB and LBW increased with maternal age over 35 years. These findings will be beneficial to subsequently explore the causes of differences in fertility in the PRD region. Similarly, the results from the Austrian Register of Births point to a higher rate of PTB in mothers aged 35 and above [47]. In relative terms, mothers of advanced age were more likely to deliver PTB and LBW babies than younger mothers.

## Strength and limitations

Our study had several strengths. This study utilized a large population-based data set. The major strengths of this study include the large sample size offering a design with minimal selection bias and the overall high-quality registry data, where almost 87% of parturients are migrants. Even so, 2.9% (*n* = 158,029) of all deliveries had missing or incorrect information. The study also has some limitations. There were many important factors we did not cover in this study as a limitation of the database. The birth certificate lacks data about maternal biometrics, pre-pregnancy clinical comorbidities, health behavior, utilization of maternity health care, health care insurance and duration of residence for migrant women. The study also lacks data on detailed characteristics of socioeconomic factors, such as household income, work status, or marital/ relationship status, education status which prevents further study of these groups of migrants.

## Conclusion

In conclusion, our study is one of the few studies based on a large cohort of newborns to understand the relationship between the migration of women and birth outcomes in China. When comparing the perinatal outcome of childbearing migrants and local women, a more favorable profile was observed among migrants. The findings of this study contribute to the understanding of the epidemiology of PTB and LBW among migrants. Our study suggests that it is the health and robust nature of migrant mothers that predisposes them to better birth outcomes. Further studies are needed to more deeply explore issues related to migration history and more detailed characteristics of the socioeconomic factors of mothers.

## Data Availability

No datasets were generated or analysed during the current study.

## Declarations

### Patient and public involvement

Patients and/or the public were not involved in the design, conduct, reporting or dissemination plans of this research. Patient consent for publication Not required.

## Abbreviations

PRD: China’s Pearl River Delta Region
PTB: preterm birth
LBW: low birth weight
